# Vertical Transmission of Zika Virus by *Aedes aegypti* and *Ae. albopictus* Mosquitoes

**DOI:** 10.3201/eid2305.162041

**Published:** 2017-05

**Authors:** Alexander T. Ciota, Sean M. Bialosuknia, Dylan J. Ehrbar, Laura D. Kramer

**Affiliations:** Wadsworth Center, New York State Department of Health, Slingerlands, New York, USA (A.T. Ciota, S.M. Bialosuknia, D.J. Ehrbar, L.D. Kramer);; State University of New York, School of Public Health, Albany, New York, USA (A.T. Ciota, L.D. Kramer)

**Keywords:** arbovirus infections, Zika virus, vertical transmission, *Aedes* mosquitoes, viruses, vector-borne infections

## Abstract

To determine the potential role of vertical transmission in Zika virus expansion, we evaluated larval pools of perorally infected *Aedes aegypti* and *Ae. albopictus* adult female mosquitoes; ≈1/84 larvae tested were Zika virus–positive; and rates varied among mosquito populations. Thus, vertical transmission may play a role in Zika virus spread and maintenance.

Following the 2007 outbreak in Micronesia, Zika virus (*Flaviviridae, Flavivirus*) has continued to expand its distribution throughout the Pacific region and, since 2014, the Americas (*1*,*2*). The virus is primarily maintained by horizontal transmission between *Aedes aegypti* mosquitoes and humans, yet other *Aedes* spp. are also competent vectors (*3*). The extent to which Zika virus can utilize vertical transmission between mosquitoes (i.e., transmission from an infected adult female mosquito to her progeny) has not been adequately assessed after peroral infection. Such studies are required to accurately determine the potential role of vertical transmission in Zika virus expansion and maintenance.

Although previous studies have found that other flaviviruses, including West Nile (*4*), dengue (*5*), yellow fever (*6*), and St. Louis encephalitis (*7*), can undergo vertical transmission, such transmission is generally relatively inefficient, with filial infection rate (FIR) estimates ranging from 1/36 to 1/6,400 (*8*). A previous study estimated rates for Zika virus vertical transmission in *Ae. aegypti* mosquitoes to be 1/290, yet a reliable estimate for transmission in *Ae. albopictus* mosquitoes was not achieved (*8*). In addition, these estimates were based on intrathoracic inoculation of Zika virus rather than assessment after infectious blood meal acquisition.

We exposed laboratory colonies of *Ae. aegypti* mosquitoes (collected in Posadas, Argentina, or Poza Rica, Mexico) and *Ae. albopictus* mosquitoes (obtained from Suffolk County, New York) to Zika virus through infectious blood meals and evaluated the mosquitoes’ capacity to transmit the virus to progeny. For this study, we used the Zika virus strain ZIKV HND (Honduras 2016, GenBank accession no. KX906952), passaged once on C6/36 cells, and Zika virus PR (Puerto Rico 2015, GenBank accession no. KX087101.3), passaged 4 times on Vero cells and twice on C6/36 cells. Zika virus was propagated on C6/36 cells for 4 days, and freshly harvested supernatant was mixed 1:1 with sheep blood (Colorado Serum Company, Denver, CO, USA) and 2.5% sucrose.

Infectious blood meals were offered to 4- to 7-day-old female mosquitoes, and weekly noninfectious blood meals were offered after the first oviposition. Eggs laid during the second oviposition and beyond were collected and hatched for subsequent testing. Third- to fourth-instar larvae were collected in pools of 5 and processed by homogenization and centrifugation. After RNA extraction, we used Zika virus–specific quantitative reverse transcription PCR (*9*) to determine adult infection (indicated by positive bodies), dissemination (indicated by positive legs), viral load, and FIR, which was calculated by using a maximum-likelihood estimate (PoolInfRate 4.0; Centers for Disease Control and Prevention, Atlanta, GA, USA).

We tested 104 *Ae. aegypti* pools; 6 were Zika virus–positive, indicating a FIR of 11.9 (range 4.9–24.6; Table). This value equates to a ratio of ≈1:84, which is substantially higher than that found by Thangamani et al., as well as ratios historically measured for flaviviruses (*4*–*8*). Although just 17 pools of *Ae. albopictus* were tested, 1 pool was positive, which equates to a similar FIR (11.8 [range 1.7–134.8]; Table) and establishes that *Ae. albopictus* mosquitoes are capable of vertical transmission of Zika virus in the laboratory.

**Table:** Vertical transmission of Zika virus in *Aedes* spp. mosquitoes*

Species/ population	Zika virus strain	Cycle	Blood meal titer, log_10_ PFU/mL	% Infected (diss)†	Mean body titer, log_10_ PFU/mL	Total no. pools	No. individual mosquitoes	dpi	Zika virus positive	FIR‡ (95% CI)
*Ae. aegypti*										
Mexico	ZIKV HND	All§	8.9	90.9 (95.0)	7.6	26	130	11–18	1	7.7 (0.5–36.9)
Argentina	ZIKV HND	All§	9.3	100 (100)	6.6	28	136	11–38	2	14.9 (2.7–8.3)
Combined¶	ZIKV HND	OV2				29	141	11–22	1	7.1 (0.4–4.0)
		OV3				23	115	18–38	2	17.7 (3.2–57.2)
		OV4				2	10	38	0	<94.6 (6.6–495.8)
Combined¶	ZIKV HND	All§	9.1#	95.5 (97.5)	7.4	54	266	11–38	3	11.5 (3.0–30.8)
Argentina	ZIKV PR	OV1				24	120	36–38	2	17.0 (3.1–54.8)
		OV2				15	75	43–52	0	<13.3 (0.8–63.6)
		OV3				4	18	60–62	0	<55.8 (3.4–262.5)
		OV4				7	35	63	1	28.5 (1.7–34.8)
	ZIKV PR	All§	9.1	100 (100)	7.7	50	248	36–63	3	12.3 (3.3–33)
Combined	Combined**	All§	9.1#	96.9 (98.3)	7.5	104	514	11–63	6	11.9 (4.9–4.6)
*Ae. albopictus*										
New York	ZIKV HND	All§	8.9	100 (93.3)	7.1	17	85	11–63	1	11.8 (0.7–56.2)

*Diss, disseminated; dpi, days post infection; FIR, filial infection rate; ZIKV HND, Zika virus Honduras 2016; OV, oviposition; ZIKV PR, Zika virus Puerto Rico. †Percentage of infected with Zika virus–positive legs. ‡No. Zika virus positive/1,000 larvae. §Combines data from all hatched eggs. ¶Data for both mosquito populations are combined. #Mean titer. **Data for both virus strains are combined.

Although the bypassing of the midgut during inoculation generally results in higher levels of vertical transmission, we fed mosquitoes high virus doses (8.9–9.3 log_10_ PFU/mL), resulting in >93% of disseminated infections and development of high viral titers in individual mosquitoes, averaging 7.1 (*Ae. albopictus*) to 7.5 (*Ae. aegypti*) log_10_ PFU/mosquito (Table). Although the likelihood that eggs were derived from mosquitoes with disseminated infections is high, the rate of vertical transmission (proportion of infected mosquitoes transmitting to progeny) could not be determined. Future studies assessing infection status and FIR of individual mosquitoes will help clarify the extent of individual variability in vertical transmission efficiency. In addition, we tested larvae rather than adults, and it is likely that transtadial transmission is not completely efficient, so further studies are required to fully evaluate transmission potential of adults infected via vertical transmission. We observed a trend of increasing vertical transmission with time and additional egg laying, similar to what has been reported for West Nile virus (*10*). This finding suggests that survival and gonotropic cycles could be key determinants of success of vertical transmission in nature. Finally, our results demonstrate population-specific differences, with the FIR of the population from Argentina more than twice that of the population from Mexico (Table), suggesting that particular populations may have increased capacity for maintenance through vertical transmission. Although we did not measure differences between ZIKV HND and ZIKV PR, evaluating additional strains could help clarify the influence of viral genotype on vertical transmission efficiency.

Together, these results indicate that Zika virus has a relatively high capacity for being transmitted vertically by both *Ae. aegypti* and *Ae. albopictus* mosquitoes. Although the mechanism of vertical transmission with flaviviruses is generally thought to be infection of eggs during oviposition, rather than transovarial transmission (*5*), these rates suggest that further investigation into Zika virus tropism in mosquitoes is warranted.
